# Supplementary material for: Hybrid Pressure Sensor Based on Carbon Nano-Onions and Hierarchical Microstructures with Synergistic Enhancement Mechanism for Multi-Parameter Sleep Monitoring
Source: Nanomaterials (Basel). 2023 Oct 1;13(19):2692. doi: 10.3390/nano13192692 (PMC10574041; doi:10.3390/nano13192692)
Supplement: Supplementary file 1 [file nanomaterials-13-02692-s001.zip › nanomaterials-2610263-supplementary.pdf]

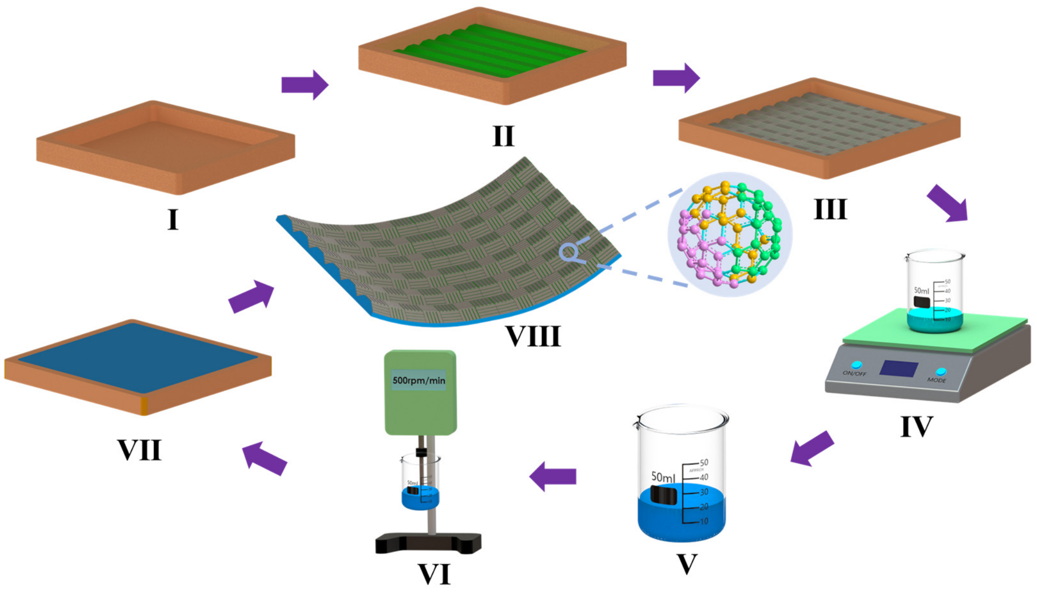

Figure S1. The detailed preparation procedure for CNOs@Ecoflex.

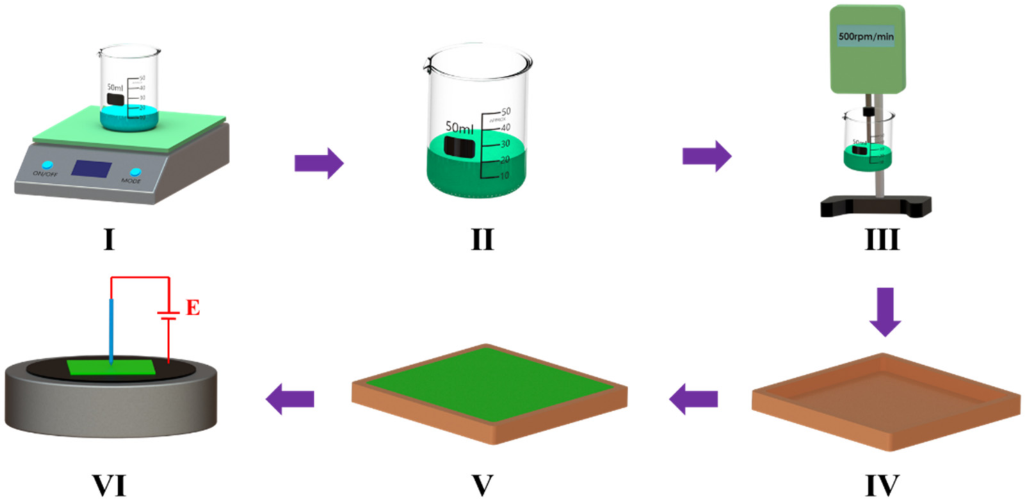

Figure S2. The detailed preparation procedure for BTO@Ecoflex.

Preparation of BTO@Ecoflex: In the first step, the mold was prepared by 3D printing. In the second step, Ecoflex component A and BTO were added into a beaker and stirred with an electric mixer for about 3-5 h. After that, Ecoflex component B was added and continued to be stirred for a period of time to form a mixed solution. In the third step, the mixed solution was poured into the prepared mold and left at room temperature for one day and then peeled off, at which time, the preparation of BTO@Ecoflex was completed. In the fourth step, the prepared BTO@Ecoflex was polarized. In the fifth step, the polarized BTO@Ecoflex was cut into the required shape.

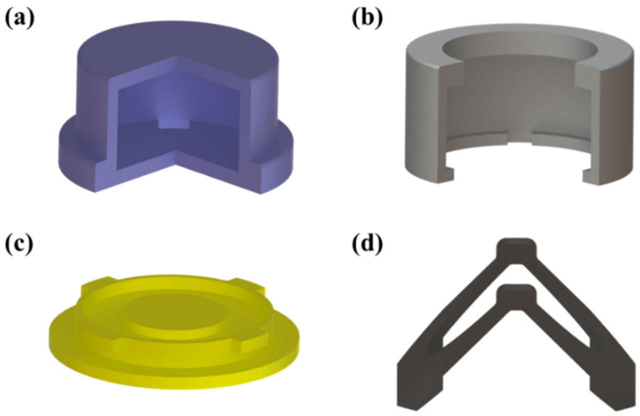

Figure S3. Structure and photograph of various structural components of HPS.

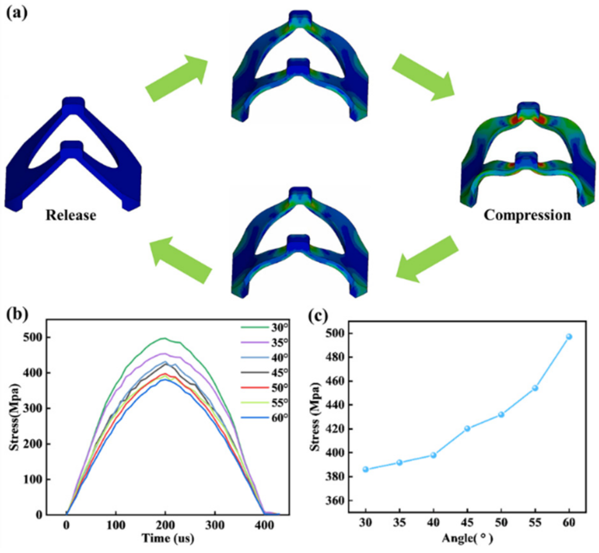

Figure S4. Maximum pressure on butterfly mechanisms with different θ.

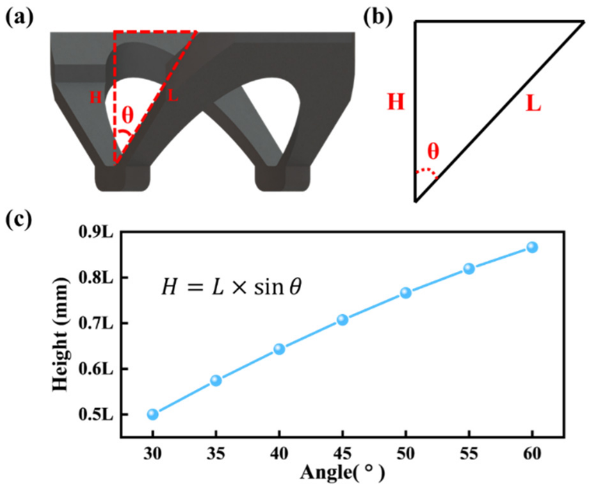

Figure S5. Height of butterfly mechanisms with different θ.

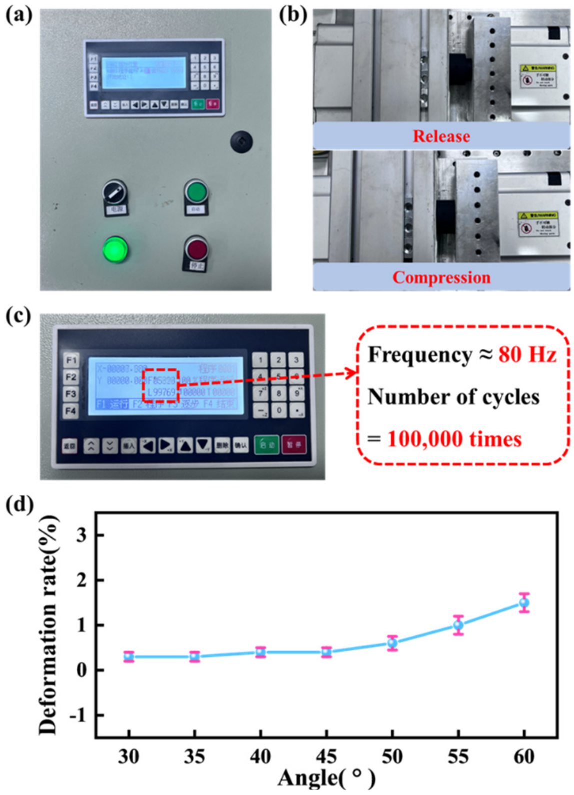

Figure S6. Durability experiment of butterfly mechanisms with different θ.

The experiments on the mechanical characteristics of the butterfly mechanism include the following three parts: (1) Figure S4 depicts the maximum pressure subjected in butterfly mechanisms with different θ in the operating process. The experiments will be implemented through ANSYS simulation software. Figure S4a depicts the pressure variations that the butterfly mechanism is subjected to at different stages (Video S1), which is described extensively the working principle of HPS in the manuscript. The simulation of ANSYS software can be found to obtain the variation of the pressure that the butterfly mechanism is subjected to in the operation, and the specific results are shown in Figure S4b. Figure S4c demonstrates the maximum pressure that the butterfly mechanism with different θ is subjected to during the compression experiment. (2) The height of the butterfly mechanism is closely related to the electrical output of the TENG, and Figure S5 illustrates the maximum heights of the butterfly mechanisms with different θ. With the hypotenuse L constant, θ of the butterfly mechanism is proportional to H. (3) Figure S6 illustrates the durability experiments of the butterfly mechanism. Figures S6a-c describe the experimental platform for the compression experiment, where the number of cycles and the compression frequency can be adjusted through the control panel. The number of cycles for the compression experiment was 100,000, and the compression frequency was roughly 80 Hz. Compression experiments were performed on butterfly mechanisms with different θ, respectively. The deformation rate of the butterfly mechanism was tested at the end of the experiment and the results are shown in Figure S6d.

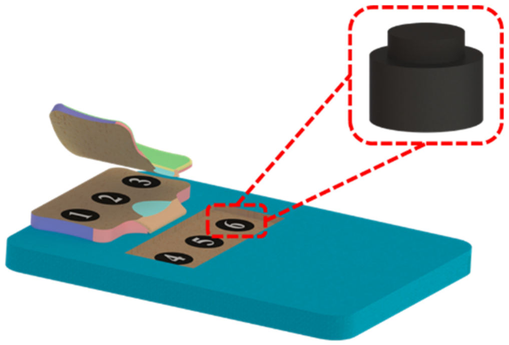

Figure S7. Schematic diagram of the sleep monitoring system with integrated HPS.

In terms of biocompatibility, HPS designed in the manuscript will be integrated into pillows and mattresses for breathing and sleeping posture monitoring. Nylon is chosen for the shell of HPS. Nylon is a non-toxic, environmentally friendly material that is not damaging to human beings. Its excellent mechanical properties make HPS more stability and environmentally adaptable. In addition, HPS is integrated into the bottom of the pillow and mattress, and there is a layer of foam above HPS. Therefore, the non-flexible shell does not greatly affect human sleep comfort. Therefore, HPS designed in the manuscript is excellent in biocompatibility.

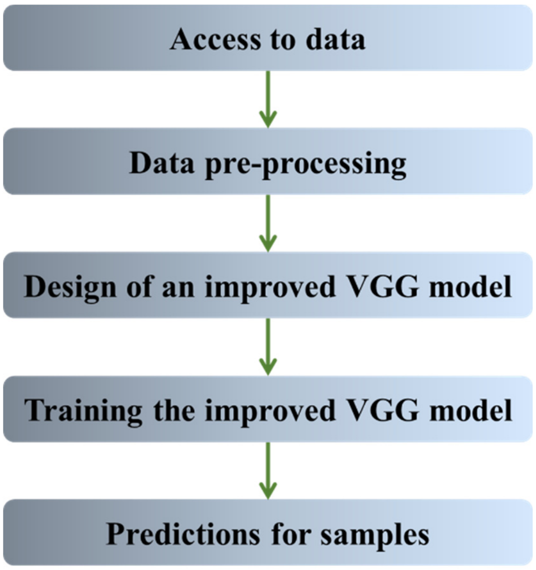

Figure S8. Detailed flow of prediction algorithms for different breathing states.

The prediction process for different breathing conditions is as follows. (1) Acquire the respiration curves of volunteers in different respiratory condition and save them. (2) Preprocess the acquired data, which mainly includes outliers value processing and dataset production. The dataset production includes data cutting, overlapping sampling (dataset enhancement) and splitting the training set and test set. (3) Design the improved VGG model, based on the VGG model, the following improvements are made: improve the first layer of the large convolutional kernel, reduce the number of neurons in the fully connected layer, and adopt the mean pooling process. (4) The improved VGG model is trained with the adam training algorithm, which dynamically adjusts the learning rate of each parameter using the first-order matrix estimation and second-order matrix estimation of the gradient. (5) Predict the prediction set by the

improved VGG model, and finally obtain the identification results of different breaths.

Table S1 Comparison of existing sleep monitoring systems with the sleep monitoring system in this work.

Sensor Type	Number of sensor	Types of Breathing	accuracy of breathing recognition	Types of posture	accuracy of posture recognition	Reference
Pressure sensor	4	2	/	3	/	[11]
Pressure sensitive sheet	1024	/	/	6	91.24%	[19]
Accelerometer and pressure sensor	2	2/4	95.1%/79.7%	/	/	[20]
Pressure sensor	8192	/	/	14	91.21%	[21]
Pressure sensor	1	4	92.5%	/	/	[22]
piezoelectric polymer	8192	/	/	5	90.3%	[23]
Pressure sensor	6	4	100%	5	/	This work

A detailed comparison was made between the existing sleep monitoring system and the one in the manuscript, the results of the comparison are shown in Table S1. The sleep monitoring system designed in this work has the functions of both breathing monitoring and sleeping posture monitoring, and it has certain advantages in terms of recognition types and accuracy. The sleep monitoring system designed in this work contributes to the development of the sleep monitoring field.

References

11. Adepu, V.; Kamath, K.; Mattela, V.; Sahatiya, P. Development of Ti3C2Tx/NiSe2 Nanohybrid-Based Large-Area Pressure Sensors as a Smart Bed for Unobtrusive Sleep Monitoring. *Adv. Mater. Interfaces* **2021**, 8, 2100706. [CrossRef]

19. Hu, Q.S.; Tang, X.C.; Tang, W. A Real-Time Patient-Specific Sleeping Posture Recognition System Using Pressure Sensitive Conductive Sheet and Transfer Learning. *IEEE Sens. J.* **2021**, 21, 6869–6879. [CrossRef]

20. He, C.H.; Tan, J.W.; Jian, X.L.; Zhong, G.X.; Cheng, L.L.; Lin, J.Z. A Smart Flexible Vital Signs and Sleep Monitoring Belt Based on MEMS Triaxial Accelerometer and Pressure Sensor. *IEEE Internet Things J.* **2022**, 9, 14126–14136. [CrossRef]

21. Xu, X.W.; Lin, F.; Wang, A.S.; Hu, Y.; Huang, M.C.; Xu, W.Y. Body-Earth Mover's Distance: A Matching-Based Approach for Sleep Posture Recognition. *IEEE Trans. Biomed. Circuits Syst*. **2016**, 10, 1023–1035. [CrossRef]

22. Shin, J.H.; Chee, Y.J.; Jeong, D.U.; Park, K.S. Nonconstrained Sleep Monitoring System and Algorithms Using Air-Mattress with Balancing Tube Method. *IEEE Trans. Inf. Technol. Biomed*. **2010**, 14, 147–156. [CrossRef]

23. Huang, M.C.; Liu, J.J.; Xu, W.Y.; Alshurafa, N.; Zhang, X.Y.; Sarrafzadeh, M. Using Pressure Map Sequences for Recognition of on Bed Rehabilitation Exercises. *IEEE J. Biomed. Health Inform.* **2014**, 18, 411–418. [CrossRef]
